# The effect of alkyl substitution on the oxidative metabolism and mutagenicity of phenanthrene

**DOI:** 10.1007/s00204-022-03239-9

**Published:** 2022-02-19

**Authors:** Danlei Wang, Viktoria Schramm, Jeroen Pool, Eleni Pardali, Annemarijn Brandenburg, Ivonne M. C. M. Rietjens, Peter J. Boogaard

**Affiliations:** Division of Toxicology, Wageningen University and Research, 6708 WE Wageningen, The Netherlands

**Keywords:** Alkylated phenanthrene, Michaelis–Menten kinetics, Rat, Human, Microsomes, Mutagenicity

## Abstract

Alkyl-substituted PAHs may be present in certain petroleum-derived products and in the environment and may eventually end up in consumer products, such as foodstuffs, cosmetics and pharmaceuticals. Safety concerns over possible exposure to alkylated PAHs have emerged. Bioactivation is a prerequisite for the mutagenicity and carcinogenicity of PAHs and has been extensively studied for non-substituted PAHs, while data on the bioactivation of alkyl-substituted PAHs are scarce. The present study investigated the effect of alkyl substitution on the CYP 450-mediated metabolism of phenanthrene and eight of its alkylated congeners by quantifying metabolite formation in rat and human liver microsomal incubations. Furthermore, the mutagenicity of four selected methylated phenanthrenes was compared to that of phenanthrene using the Ames test. The obtained results support the hypothesis that alkyl substitution shifts the oxidative metabolism from the aromatic ring to the alkyl side chain. Increasing the length of the alkyl chain reduced overall metabolism with metabolic conversion for 1-n-dodecyl-phenanthrene (C12) being negligible. 1- and 9-methyl-phenanthrene, in which the methyl group generates an additional bay region-like structural motif, showed mutagenicity toward *Salmonella typhimurium* TA98 and TA 100, whereas phenanthrene and also 2- and 3-methyl-phenanthrene, without such an additional bay region-like structural motif, tested negative. It is concluded that the position of the alkylation affects the metabolism and resulting mutagenicity of phenanthrene with the mutagenicity increasing in cases where the alkyl substituent creates an additional bay region-like structural motif, in spite of the extra possibilities for side chain oxidation.

**Supplementary Information:**

The online version contains supplementary material available at 10.1007/s00204-022-03239-9.

## Introduction

The unintentional consumption of polycyclic aromatic hydrocarbons (PAHs) via contaminated food is a general food safety issue. The PAHs present in food are typically unsubstituted as they are pyrogenic in nature, formed by incomplete heating or combustion of organic matter (EFSA 2008). However, contamination of food with petrogenic polycyclic aromatic hydrocarbons (PPAHs), which are typically alkylated, is an emerging health concern (EFSA 2012; Fengler and Gruber 2020; Grob 2018; Pirow et al. 2019; Van Heyst et al. 2018). In spite of the fact that petroleum-derived mineral oils are highly refined to eliminate undesirable substances such as mutagenic PAHs to make them compliant to EU regulations that forbid selling of carcinogenic substances to the general public (Carrillo et al. 2019), concerns about potentially carcinogenic constituents have been raised. It is known that some non-substituted and methylated polycyclic aromatic hydrocarbons with three to seven fused rings are mutagenic and potentially carcinogenic (Carrillo et al. 2019). Consumer products may be contaminated with PPAHs as a result of inappropriate use of mineral oils or via environmental contamination. For instance, crude oil spills, such as the Deepwater Horizon oil spill in the Gulf of Mexico, released large amounts of PPAHs into the environment (Fernando et al. 2019). In the Deepwater horizon oil spill phenanthrene and its methylated congeners were found to be among the most abundant PPAHs (NIST 2012). Part of these PPAHs may be taken up by marine species and end up in the food chain via consumption of seafood which has raised concerns for human health (Pulster et al. 2020; Ylitalo et al. 2012). The current knowledge on the possible metabolic activation and genotoxicity of PAHs primarily relates to unsubstituted PAHs, which are typically of pyrogenic origin. However, the potential bioactivation due to oxidative metabolism of substituted PAHs, such as PPAHs, has not been systematically investigated.

Some unsubstituted PAHs, such as benzo[a]pyrene and dibenzo[a,l]pyrene, are bioactivated by oxidation at the “bay region” or “fjord region” to highly reactive and mutagenic dihydrodiol-epoxides which may form adducts to DNA and eventually induce mutations and cancer (Boogaard 2012; Lehr et al. 1985; Tsang and Griffin 1979; Whalen et al. 1978). Information on the metabolism of PPAHs and their potential bioactivation is limited.

In our previous study, the in vitro hepatic biotransformation of naphthalene and alkyl-substituted naphthalenes was quantified (Wang et al. 2020). It was found that alkyl substitution of naphthalene shifts metabolism toward alkyl side chain oxidation at the cost of aromatic ring oxidation. To get more insight into the metabolic transformation of alkyl-substituted PAHs that may be present in mineral oils and crude oils, in the present study, the oxidative metabolism of phenanthrene and a series of its alkylated congeners is investigated.

**Fig. 1:**
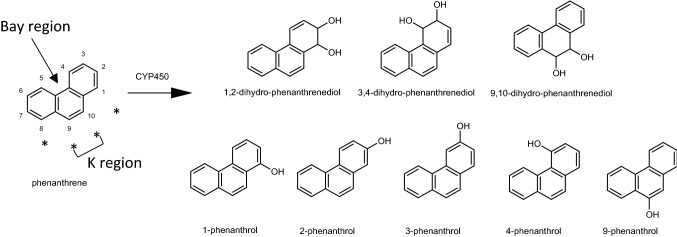
Reported metabolites of phenanthrene formed in microsomal incubations by P450 enzymes of humans and rodents (Bao and Yang 1991; Chaturapit and Holder 1978; Jacob et al. 1996; Schober et al. 2010; Shou et al. 1994). *Indicates a peri position

Phenanthrene, the smallest PAH with a bay region, was classified by IARC as group 3 (not classifiable as to its carcinogenicity to humans), based on inadequate data in experimental animals (IARC, 2010). Phenanthrene can be metabolized by cytochrome P450 enzymes from humans or rodents to 1,2-dihydrodiol-, 3,4-dihydrodiol- and 9,10-dihydrodiol-phenanthrene, and 1-, 2-, 3-, 4- and 9-phenanthrols (Fig. 1) (Bao and Yang 1991; Chaturapit and Holder 1978; Jacob et al. 1996; Schober et al. 2010; Shou et al. 1994). However, no phenanthrene DNA adducts could be detected in Chinese hamster bone marrow cells following in vivo exposure to phenanthrene (Bayer 1978). Limited data on the metabolism of alkylated congeners of phenanthrene are available. Side chain hydroxylation was reported as the major pathway of 1-methylphenanthrene and 9-ethylphenanthrene in human HepG2 cells (Huang et al. 2017). Dihydrodiols of 1-hydroxymethyl-phenanthrene, dihydrodiols of 1-methyl-phenanthrene, 1-hydroxymethyl-phenanthrene and 1-methylphenananthrenols were found in incubations with rat S9 fractions and 1-methylphenanthrene (LaVoie et al. 1981). A similar metabolite profile was reported for 9-methylphenanthrene (LaVoie et al. 1981). Metabolic patterns and potential preferences for alkyl side chain oxidation or aromatic ring oxidation of other alkylated phenanthrenes, especially the ones with longer alkyl chains, are still unclear.

The model compounds included in the present study were phenanthrene, 1-methylphenanthrene, 2-methylphenanthrene, 3-methylphenanthrene, 9-methylphenanthrene, 2-ethylphenanthene, 9-ethylphenanthrene, 10-methyl-9-ethylphenanthrene, 1-n-hexylphenanthrene and 1-n-dodecylphenanthrene. The metabolic profile, kinetics and catalytic efficiency of the conversion of these model compounds were characterized in reactions likely mediated by cytochrome P450 in rat or human liver microsomes to better define the metabolic consequences of alkylation. In addition, some selected methylated congeners as well as unsubstituted phenanthrene were tested for their mutagenicity in the Ames test, to obtain further insight into the effect of the introduction of an additional bay region-like structural motif as a result of alkylation on the mutagenicity.

## Material and methods

### Chemicals and reagents

Phenanthrene (≥ 98%), 2-ethylphenanthrene (≥ 98%), 9-ethylphenanthrene (≥ 98%), 10-methyl-9-ethylphenanthrene (≥ 98%), 3-(hydroxymethyl)-phenanthrene (≥ 95.0%), 9-(hydroxymethyl)-phenanthrene (≥ 95.0%), tetrahydrofuran (≥ 99.9%), trifluoroacetic acid (≥ 99%), methylmethanesulfonate, 2-aminoanthracene, and nitrofluorene were purchased from Sigma-Aldrich (St.Louis, USA). 1-Methylphenanthrene (≥ 96%) was obtained from Toronto Research Chemicals (North York, Canada). 3-Methylphenanthrene (≥ 98%), 1-phenanthrol (≥ 98%), 3-phenanthrol (≥ 98%), 4-phenanthrol (≥ 98%) and NADPH were supplied by Carbosynth (Berkshire, UK). 9-Methylphenanthrene (≥ 98%) and 2-methylphenanthrene (≥ 98%) were purchased from BOC Sciences (Hamburg, Germany). 1-n-Hexylphenanthrene (≥ 99.2%) and 1-n-dodecylphenanthrene (≥ 99.5%) were synthesized by the Biochemical Institute for Environmental Carcinogens (Groβhansdorf, Germany). Acetonitrile was bought from Biosolve (Dieuze, France). Dimethyl sulfoxide (DMSO), K_2_HPO_4_∙3H_2_O, MgCl_2_, and KCl were supplied by Merck (Darmstadt, Germany). Gentest™ pooled male Sprague–Dawley rat liver microsomes (RLM) and Ultrapool™ human liver microsomes (HLM) with a protein concentration of 20 mg/ml were obtained from Corning (New York, USA), and the latter contained cytochrome P450 liver enzymes of 150 individuals. Rat liver S9 homogenate was obtained from Trinova Biochem GmbH (Giessen, Germany) and was prepared from the livers of male Sprague–Dawley rats that had been injected intraperitoneally with Aroclor 1254 (500 mg/kg body weight). *Salmonella typhimurium* TA98 and TA 100 tester strains were also obtained from Trinova Biochem GmbH (Giessen, Germany). NADP and glucose-6-phosphate were supplied by Randox Laboratories Ltd. (Crumlin, UK) and Roche Diagnostics (Mannheim, Germany), respectively.

### In vitro incubations of phenanthrene and its alkylated congeners with rat and human liver microsomes

Microsomal oxidation of phenanthrene and its alkylated congeners by RLM and HLM was investigated in an overall 200 μl incubation system consisting of potassium phosphate (0.1 M, pH 7.4), containing 5 mM MgCl_2_, RLM/HLM at a final microsomal protein concentration of 0.5 mg/ml, 1 mM NADPH, and each of the individual test compounds at concentrations ranging from 0 to 600 μM. Test compounds were phenanthrene, 1-methylphenanthrene, 2-methylphenanthrene, 3-methylphenanthrene, 9-methylphenanthrene, 2-ethylphenanthrene, 9-ethylphenanthrene, 10-methyl-9-ethylphenanthrene, 1-n-hexylphenanthrene and 1-n-dodecyl-phenanthrene. The final concentration of substrate solvent, either DMSO or tetrahydrofuran (the latter was used for 1-n-hexylphenanthrene and 1-n-dodecylphenanthrene due to their low solubility in DMSO) in the incubation mixture was 1% (*v*/*v*), which did not affect the enzymatic activity of liver microsomes (Li et al. 2010). The incubation mixtures were prepared and incubated in glass vials to avoid plastic binding of the substrates. The glass vials were capped to prevent substrate loss due to volatility. After preincubation of the incubation mixture at 37 °C for 1 min, the enzymatic reaction was initiated by adding microsomes to the incubation mixture which was subsequently incubated at 37 °C for 20 min. The reaction was terminated by adding 100 µl ice-cold acetonitrile followed by vortexing. After 5 min centrifugation at 5000 rpm (4000 × *g*), 4 °C, the supernatant was collected for ultraperformance liquid chromatography (UPLC) analysis. However, the concentrations of the metabolites of two of the test substrates, 1-n-hexyl-phenanthrene and 1-n-dodecyl-phenanthrene, in the supernatant appeared too low to detect metabolism. Therefore, a diisopropylether (DIPE) extraction of the metabolites was performed after the reaction was stopped by the addition of 20 µl 10% HClO_4_. To this end, the incubation mixture (total volume of 220 µl) was extracted three times with 1 ml DIPE. Each time, the upper organic layer was collected and the DIPE was subsequently evaporated from the combined organic fractions with a gentle stream of nitrogen. The residues were dissolved in 100 µl methanol and analyzed by UPLC.

To prepare samples for metabolite identification by GC–MS/MS, each individual test substance (200 µM final concentration) was incubated in a volume of 400 μl in potassium phosphate (0.1 M, pH 7.4) containing 5 mM MgCl_2_, RLM or HLM at a final microsomal protein concentration of 1 mg/ml, and 1 mM NADPH at 37 °C for 10 min. After incubation, the incubation mixture was centrifuged for 5 min at 5000 rpm (4000 × *g*) and 4 °C. The supernatant of the incubation mixture was transferred to a fresh vial and extracted three times with 100 µl dichloromethane (DCM) following vortexing. The DCM phase containing the substrate and metabolites was separated from the aqueous phase by centrifugation at 5000 rpm (4000 × *g*) for 5 min. The organic (lower) phase was collected from each extraction, combined and analyzed by GC–MS/MS.

### Reverse mutation assay

The mutagenicity of phenanthrene and four of its methylated analogs (1-methyl-, 2-methyl-, 3-methyl- and 9-methyl-phenanthrene) was assessed in the Ames test, a bacterial reverse mutation assay, using the TA98 and TA100 strains of *S. typhimurium*. Six concentrations of each compound were tested in triplicate in the absence and presence of 5% (*v*/*v*) S9-mix prepared from the livers of Aroclor 1254 treated Sprague–Dawley rats. The S9-mix contained 4 mM NADP, 5.8 mM glucose-6-phosphate, 0.1 M sodium phosphate pH 7.4, 8 mM MgCl_2_, 33 mM KCl and 5% S9 homogenate. Fresh bacterial cultures were prepared overnight to reach 10^9^ cells/ml. The following solutions were pre-incubated in a rotating incubator at 70 rpm and 37 °C, and contained either 0.5 ml S9-mix (in case of S9 presence) or 0.5 ml 0.1 M potassium phosphate pH 7.4 (in case of S9 absence), 0.1 ml of a fresh bacterial culture (10^9^ cells/ml) of TA98 or TA100, and 10–100 µg test compound. Top agar was melted and heated to 45 °C. After preincubation, the substrate solutions were added to 3 ml of the molten top agar and mixed by vortexing and the top agar mixture was poured onto a minimal glucose agar plate. After solidification of the top agar, the plates were incubated at 37 °C for 48 h. The number of revertant colonies per plate was automatically counted with the Instem Sorcerer Colony Counter (Staffordshire, UK). In the absence of S9-mix, 2-nitrofluorene (NF) and methylmethanesulfonate (MMS) were tested as positive controls for incubations with TA98 and TA100, respectively. In the presence of S9-mix, 2-aminoanthracene (2AA) was tested as a positive control in both TA98 and TA100. DMSO was tested as a solvent control in both tester strains. The mutagenicity of the test compounds was determined by the number of revertant colonies per plate and considered positive if the revertant number was increased compared to the historical control data and was also more than threefold or twofold higher than the controls for tester strain TA98 and TA100, respectively (Levy et al. 2019). The historical control data are presented in Table S1 in the supplementary material 1.

### In vitro incubations of phenanthrene and its methylated analogs with rat S9

Since the reverse mutation assay used an S9 metabolic system instead of microsomes, for the compounds tested in the reverse mutation assay the metabolite patterns were also characterized in incubations with rat liver S9. To this end, the incubation mixture of each compound tested in the reverse mutation assay with Aroclor 1254-treated rat liver S9 was analyzed by UPLC, while unidentified metabolites were further analyzed by GC–MS/MS. Incubation mixtures consisting of 100 µg (1000 µM final concentration) test compound, either 0.5 ml S9-mix (in case of S9 presence) or 0.5 ml 0.1 M potassium phosphate pH 7.4 (in case of S9 absence) were incubated for 48 h at 37 °C, applying the concentrations, incubation time and temperature also used in the Ames assay. After the 48-h incubation, 250 µl acetonitrile was added to the incubation mixture, followed by centrifugation at 5000 rpm (4000 × *g*) for 5 min. The supernatant was collected for UPLC analysis. For GC–MS/MS analysis, the incubation mixture was centrifuged for 5 min at 5000 rpm (4000 × *g*) and 4 °C after the 48-h incubation. The supernatant thus obtained was transferred to a fresh vial and extracted three times with 100 µl DCM following vortexing. The DCM phase, containing the substrate and its metabolites, was separated from the aqueous phase by centrifugation at 4000 × *g* for 5 min. The organic (lower) phase was collected, combined and analyzed by GC–MS/MS.

### UPLC analysis

The metabolites formed were analyzed and quantified using an Acquity UPLC system equipped with a photodiode array (PDA) detector (Waters, Milford, MA). The metabolites and their parent compound were separated on a reverse phase Acquity UPLC^®^ BEH C18 column (21 × 50 mm, 1.7 µm, Waters, Milford, MA) and detected at wavelengths ranging from 190 to 400 nm. Eluent A was nano-pure water containing 0.1% trifluoroacetic acid (*v*/*v*), and eluent B was acetonitrile containing 0.1% trifluoroacetic acid (*v*/*v*). The gradient elution started from 90% A and 10% B applied from 0.0 min to 0.5 min, which was changed to 0% A and 100% B from 0.5 to 15.5 min and then kept at 0% A and 100% B from 15.5 min to 18.5 min, changed back to 90% A and 10% B from 18.5 to 18.6 min and then maintained at the starting conditions from 18.6 min until 22 min. The total run time was 22 min using a flow rate of 0.6 ml/min. The temperature of the column was set at 40 °C and that of the autosampler at 10 °C during the UPLC analysis. The injection volume was 3.5 µl. Metabolites were quantified using their peak area at the wavelength specified in Table 1, using calibration curves of available reference compounds. Metabolites were identified by comparing their retention time (RT) and UV spectrum to those of reference chemicals on UPLC. When reference chemicals were not available, metabolite identification by gas chromatography-triple quadrupole mass spectrometry (GC–MS/MS) was performed. The minor metabolites were identified by elution time and mass spectra both on UPLC and GC–MS/MS, and by comparison with available elution and spectral information from the literature.

**Table 1:** The Michaelis–Menten parameters including *K*_*M*_, *V*_max_ and intrinsic clearance (Cl_int_) calculated as V_max_/K_M_ for formation of metabolites from alkyl-substituted phenanthrenes and phenanthrene in rat and human liver microsomal incubations (Fig. 2)

Parent compound and Metabolites	Structure	Species	*K*_*M*_ (µM)	*V*_max_ (pmol/min/mg microsomal protein)	Cl_int_ (*V*_max_/*K*_*M*_) µl/min/mg protein
**Phenanthrene RT = 8.21 min,** ***λ*** **= 251.1 nm**					
3,4-Dihydro-phenanthrene-diol RT = 2.13 min, *λ* = 260.2 nm		Human	22.6 ± 12.7	61.5 ± 6.3	2.7
		Rat	153.7 ± 72.4	10.3 ± 1.8	0.1
3,4-Dihydro-phenanthrene-diol RT = 2.24 min, *λ* = 237.1 nm		Human	8.1 ± 3.5	6.0 ± 0.3	0.7
		Rat	ND	ND	–
9,10-Dihydro-phenanthene-diol RT = 3.28 min, *λ* = 231.6 nm		Human	ND	ND	–
		Rat	84.2 ± 15.7	9.8 ± 0.6	0.1
9,10-Dihydro-phenanthene-diol RT = 3.38 min, *λ* = 209.1 nm		Human	32.4 ± 9.0	287.8 ± 17.1	8.9
		Rat	121.6 ± 29.1	529.2 ± 43.2	4.4
1,2-Dihydro-phenanthrene-diol RT = 3.57 min, *λ* = 237.1 nm		Human	25.7 ± 12.6	152.5 ± 14.4	5.9
		Rat	104.3 ± 26.3	79.3 ± 6.5	0.8
3-Phenanthrol RT = 6.02 min, *λ* = 252.9 nm		Human	ND	ND	–
		Rat	103.4 ± 29.5	21.3 ± 2.0	0.2
1-Phenanthrol RT = 6.31 min, *λ* = 251.7 nm		Human	21.3 ± 5.2	125.2 ± 5.4	5.9
		Rat	83.6 ± 43.1	123.9 ± 19.2	1.5
4-Phenanthrol RT = 6.55 min, *λ* = 245 nm		Human	188.1 ± 104.1	18.0 ± 3.9	0.1
		Rat	145.3 ± 89.8	24.5 ± 5.5	0.2
**1-Methylphenanthrene RT = 8.99 min,** ***λ*** **= 255.3 nm**					
Dihydro-1-methylphenanthrene-diol RT = 2.32 min, *λ* = 210.4 nm		Human	430.8 ± 134.7	27.5 ± 4.6	0.1
		Rat	ND	ND	–
Dihydro-1-methylphenanthrene-diol RT = 3.43 min, *λ* = 255.3 nm		Human	ND	ND	–
		Rat	43.7 ± 19.0	4.2 ± 0.4	0.1
Dihydro-1-methylphenanthrene-diol RT = 3.96 min, *λ* = 260 nm		Human	ND	ND	–
		Rat	17.8 ± 6.7	5.2 ± 0.3	0.3
Dihydro-1-methylphenanthrene-diol RT = 4.35 min, *λ* = 238.9 nm		Human	25.1 ± 3.3	20.6 ± 0.5	0.8
		Rat	34.3 ± 12.9	16.1 ± 1.3	0.5
Dihydro-1-methylphenanthrene-diol RT = 4.76 min, *λ* = 254.1 nm		Human	ND	ND	–
		Rat	14.8 ± 4.4	14.0 ± 0.6	0.9
1-(Hydroxymethyl) phennathrene RT = 5.55 min, *λ* = 254.1 nm		Human	30.5 ± 4.1	475.3 ± 13.2	15.6
		Rat	37.4 ± 3.5	1926 ± 40.3	51.5
1-Methylphenanthrol RT = 6.3 min, *λ* = 252.9 nm		Human	107.5 ± 18.9	14.7 ± 0.8	0.1
		Rat	ND	ND	–
1-Methylphenanthrol RT = 6.80 min, *λ* = 258 nm		Human	22.7 ± 6.2	77.3 ± 3.9	3.4
		Rat	123.0 ± 22.4	33.1 ± 2.1	0.3
1-Methylphenanthrol RT = 6.92 min, *λ* = 249.9 nm		Human	ND	ND	–
		Rat	106.5 ± 21.5	24.0 ± 1.6	0.2
1-Methylphenanthrol RT = 7.00 min, *λ* = 258 nm		Human	ND	ND	–
		Rat	93.4 ± 21.6	17.3 ± 1.2	0.2
1-Methylphenanthrol RT = 7.29 min, *λ* = 256 nm		Human	ND	ND	–
		Rat	150.2 ± 37.6	9.0 ± 0.8	0.1
**2-Methylphenanthrene RT = 9.05 min,** ***λ*** **= 252.9 nm**					
Dihydro-2-methylphenanthrene-diol RT = 3.09 min, *λ* = 262.1 nm		Human	35.9 ± 11.9	6.4 ± 0.5	0.2
		Rat	ND	ND	–
Dihydro-2-methylphenanthrene-diol RT = 4.26 min, *λ* = 212.2 nm		Human	ND	ND	–
		Rat	135.8 ± 44.5	23.1 ± 2.7	0.2
Dihydro-2-methylphenanthrene-diol RT = 4.42 min, *λ* = 243.8 nm		Human	ND	ND	–
		Rat	95.9 ± 46.6	16.2 ± 2.5	0.2
Dihydro-2-methylphenanthrene-diol RT = 4.99 min, *λ* = 252.9 nm		Human	ND	ND	–
		Rat	141.7 ± 43.3	12..8 ± 1.4	0.1
2-(Hydroxymethyl)-phennathrene RT = 5.59 min, *λ* = 253.5 nm		Human	21.6 ± 5.9	562.0 ± 27.5	26.0
		Rat	25.8 ± 7.1	2174 ± 115.3	84.3
2-Methylphenanthrol RT = 6.12 min, *λ* = 262.7 nm		Human	4.9 ± 3.2	16.3 ± 0.8	3.3
		Rat	NA	90.7 ± 3.8	–
2-Methylphenanthrol RT = 6.98 min, *λ* = 266.9 nm		Human	ND	ND	-
		Rat	15.6 ± 4.2	76.0 ± 3.1	4.9
2-Methylphenanthrol RT = 7.08 min, *λ* = 260.8 nm		Human	ND	ND	–
		Rat	39.6 ± 13.3	11.2 ± 0.9	0.3
**3-Methylphenanthrene RT = 9.04 min,** ***λ*** **= 252.3 nm**					
Dihydro-3-methylphenanthrene-diol RT = 4.25 min, *λ* = 210.4 nm		Human	100.8 ± 20.0	65.0 ± 4.1	0.6
		Rat	312.5 ± 178.2	92.3 ± 25.1	0.3
Dihydro-3-methylphenanthrene-diol RT = 4.42 min, *λ* = 238.9 nm		Human	71.5 ± 16.2	12.8 ± 0.8	0.2
		Rat	121.5 ± 29.0	20.4 ± 1.7	0.2
3-(Hydroxymethyl)-phenanthrene RT = 5.62 min, *λ* = 252.9 nm		Human	44.5 ± 6.2	583.8 ± 19.6	13.1
		Rat	120.2 ± 41.7	2593 ± 305	21.6
3-Methylphennathrol RT = 6.98 min, *λ* = 252.9 nm		Human	ND	ND	–
		Rat	371.1 ± 476.3	82.6 ± 53.8	0.2
**9-Methylphenanthrene RT = 9.02 min,** ***λ*** **= 252.9 nm**					
3,4-Dihydro-9-methylphenanthrene-diol RT = 2.98 min, *λ* = 262.1 nm		Human	55.9 ± 23.9	26.1 ± 3.0	0.5
		Rat	ND	ND	–
1,2-Dihydro-9-methylphenanthrene-diol RT = 4.27 min, *λ* = 238.9 nm		Human	61.8 ± 33.7	15.6 ± 2.4	0.3
		Rat	ND	ND	–
9-(Hydroxymethyl)-phenanthrene RT = 5.6 min, *λ* = 252.9 nm		Human	35.3 ± 16.3	415.9 ± 43.9	11.8
		Rat	89.9 ± 42.9	1543 ± 226.6	17.2
9-Methylphenanthrol RT = 6.28 min, *λ* = 249.9 nm		Human	278.3 ± 145.8	27.8 ± 6.7	0.1
		Rat	ND	ND	–
9-Methylphenanthrol RT = 6.73 min, *λ* = 252.9 nm		Human	239.8 ± 235.5	44.9 ± 19.4	0.2
		Rat	226.2 ± 99.5	27.2 ± 5.1	0.1
9-Methylphenanthrol RT = 6.87 min, *λ* = 254.7 nm		Human	ND	ND	–
		Rat	346.4 ± 164.3	124.2 ± 29.2	0.4
9-Methylphenanthrol RT = 7.2 min, 249.9 nm		Human	ND	ND	–
		Rat	445.7 ± 463.6	41.4 ± 23.4	0.1
9-Methylphenanthrol RT = 7.25 min, *λ* = 252.9 nm		Human	ND	ND	–
		Rat	154.4 ± 78.6	10.2 ± 1.9	0.1
**2-Ethylphenanthrene, RT = 9.84 min,** ***λ*** **= 253.5 nm**					
Dihydro-2-ethylphenanthrene-diol RT = 4.6 min, *λ* = 254.1 nm		Human	ND	ND	–
		Rat	25.3 ± 23.2	33.9 ± 11.8	1.3
Dihydro-2-ethylphenanthrene-diol RT = 5.16 min, *λ* = 276.6 nm		Human	108.3 ± 56.2	18.4 ± 3.2	0.2
		Rat	ND	ND	–
Dihydro-2-ethylphenanthrene-diol RT = 5.29 min, *λ* = 241.9 nm		Human	132.0 ± 49.6	22.1 ± 3.0	0.2
		Rat	181.7 ± 38.3	42.9 ± 3.5	0.2
2-(1-Hydroxyethyl)-phenanthrene RT = 6.21 min, *λ* = 253.5 nm		Human	96.5 ± 31.7	468.8 ± 50.3	4.9
		Rat	105.4 ± 27.4	3149.0 ± 266.1	29.9
2-Ethylphenanthrol RT = 7.57 min, *λ* = 255.3 nm		Human	ND	ND	–
		Rat	223.6 ± 100.8	16.5 ± 3.1	0.1
2-Ethylphenanthrol RT = 7.74 min, *λ* = 254.1 nm		Human	ND	ND	–
		Rat	233.3 ± 89.7	16.6 ± 2.7	0.1
2-Ethylphenanthrol RT = 7.89 min, *λ* = 254.1 nm		Human	ND	ND	–
		Rat	133.2 ± 76.4	11.8 ± 2.4	0.1
RT = 9.38 min, λ = 267.6 nm	Unknown	Human	205.0 ± 85.7	37.2 ± 6.5	0.2
		Rat	95.4 ± 30.5	238.0 ± 23.9	2.5
**9-Ethylphenanthrene RT = 9.71 min,** ***λ*** **= 252.9 nm**					
Dihydro-9-ethylphenanthrene-diol RT = 3.81 min, *λ* = 263.3 nm		Human	104.6 ± 17.3	15.2 ± 0.8	0.1
		Rat	117.7 ± 59.9	12.0 ± 2.1	0.1
Dihydro-9-ethylphenanthrene-diol RT = 3.88 min, *λ* = 262.1 nm		Human	59.1 ± 12.4	23.5 ± 1.4	0.4
		Rat	ND	ND	–
9-(2-Hydroxyethyl)-phenanthrene RT = 6.00 min, *λ* = 253.5 nm		Human	22.6 ± 6.4	61.4 ± 3.3	2.7
		Rat	72.9 ± 15.2	37.0 ± 2.26	0.5
9-(1-Hydroxyethyl)-phenanthrene RT = 6.20 min, *λ* = 252.9 nm		Human	122.2 ± 22.5	267.7 ± 17.4	2.2
		Rat	138.2 ± 15.2	1206 ± 47.9	8.7
9-Ethylphenanthrol RT = 7.42 min, *λ* = 252.9 nm		Human	159.9 ± 34.9	12.8 ± 1.1	0.1
		Rat	91.6 ± 30.2	29.7 ± 3.1	0.3
9-Ethylphenanthrol RT = 7.58 min, *λ* = 254.1 nm		Human	244.7 ± 52.6	29.1 ± 2.8	0.1
		Rat	321.4 ± 70.5	56.6 ± 1.8	0.2
9-Ethylphenanthrol RT = 7.93 min, *λ* = 250.5 nm		Human	ND	ND	–
		Rat	90.3 ± 23.1	22.8 ± 1.8	0.3
RT = 9.35 min, *λ* = 256.6 nm	Unknown	Human	ND	ND	–
		Rat	169.0 ± 51.7	52.7 ± 6.2	0.3
**10-Methyl-9-ethylphenanthrene RT = 10.19 min,** ***λ*** **= 255.3 nm**					
Dihydro-10-methyl-9-ethylphenanthrene-diol RT = 4.29 min, *λ* = 266.3 nm		Human	141.8 ± 83.7	7.2 ± 1.7	0.1
		Rat	253.1 ± 223.1	6.4 ± 2.7	0.0
Dihydro-10-methyl-9-ethylphenanthrene-diol RT = 4.34 min, *λ* = 266.3		Human	102.1 ± 70.3	10.5 ± 2.5	0.1
		Rat	73.7 ± 45.0	8.09 ± 1.6	0.1
9-(2-Hydroxyethyl)-10-methylphenanthrene RT = 6.5 min, *λ* = 255.3 nm		Human	24.7 ± 16.5	12.9 ± 1.8	0.5
		Rat	88.4 ± 39.2	21.0 ± 3.1	0.2
9-(1-Hydroxyethyl)-10-methylphenanthrene RT = 6.65 min, *λ* = 256 nm		Human	192.4 ± 106.8	187.1 ± 45.2	1.0
		Rat	114.3 ± 4.5	630.2 ± 81.4	5.5
9-Ethyl-10-(1-hydroxymethyl)-phenanthrene RT = 6.90 min, *λ* = 256 nm		Human	118.2 ± 65.0	85.9 ± 17.4	0.7
		Rat	98.9 ± 38.5	85.8 ± 11.6	0.9
9-Ethyl-10-methylphenanthrol RT = 7.82 min, *λ* = 255.3 nm		Human	ND	ND	–
		Rat	94.4 ± 41.4	65.6 ± 9.8	0.7
9-Ethyl-10-methylphenanthrol RT = 8.3 min, *λ* = 252.3 nm		Human	ND	ND	–
		Rat	219.8 ± 126.8	29.4 ± 7.7	0.1
**1-N-hexylphenanthrene RT = 12.60 min,** ***λ*** **= 256 nm**					
1-Hydroxyhexyl-phenanthrene RT = 8.45 min, *λ* = 256 nm		Human	67.9 ± 20.0	48.3 ± 6.1	0.7
		Rat	35.8 ± 18.1	103.4 ± 18.1	2.9
1-Hydroxyhexyl-phenanthrene RT = 8.58 min, *λ* = 256 nm		Human	ND	ND	–
		Rat	42.2 ± 10.9	54.8 ± 5.2	1.3
1-Hydroxyhexyl-phenanthrene RT = 8.82 min, *λ* = 256 nm		Human	51.7 ± 14.2	44.2 ± 4.8	0.9
		Rat	26.6 ± 6.0	25.6 ± 1.8	1.0
1-Hydroxyhexyl-phenanthrene RT = 9.13 min, *λ* = 256 nm		Human	ND	ND	–
		Rat	33.1 ± 7.3	11.7 ± 0.9	0.4
**1-N-dodecylphenanthrene RT = 16.62 min,** ***λ*** **= 302.5 nm**					
NM	NA	Human	NA	NA	NA
		Rat	NA	NA	NA

The retention time (RT) and wavelength (λ) used to identify and quantify the metabolites by UPLC–UV analysis are also presented. Results are shown as mean ± standard error of the mean (SEM) from three independent microsomal incubations

*ND*  not detected, *NM*  no metabolism, *NA*  not applicable

Under the conditions used metabolite formation was linear with time and the amount of microsomal protein. The metabolite concentrations in the microsomal incubation mixtures as quantified by UPLC were used to calculate the rate of the enzymatic conversions in pmol/min/mg microsomal protein. The kinetic parameters *K*_*M*_ and *V*_max_ were obtained using a nonlinear regression curve fit applying the Michaelis Menten equation in GraphPad Prism 5 (San Diego, USA). To compare the catalytic efficiency of formation of the different metabolites the intrinsic clearance (Cl_int_) was calculated as *V*_max_ divided by *K*_*M*_.

### Metabolite identification by GC–MS/MS

The MS spectra of all metabolites were recorded and compared to mass spectra from the NIST library (14, 14 s, 17–1, 17–2, 17 s) available in the GC–MS/MS solution software Version 4.45 (Shimadzu, Japan).

The metabolites formed from phenanthrene, 1-methylphenanthrene, 2-methylphenanthrene, 3-methylphenanthrene, 9-methylphenanthrene, 2-ethylphenanthrene, 9-ethylphenanthrene, 10-methyl-9-ethylphenanthrene, 1-n-hexylphenanthrene and 1-n-dodecylphenanthrene were analyzed using a Shimadzu GC–MS/MS system consisting of a GC-2010 Plus coupled with a mass spectrometer TQ8040 (Shimadzu, Japan). A 30 m capillary column with 0.25 mm diameter (ZB-1, Phenomenex, USA) was used to separate the metabolites upon injection of 1 µl of the extract with splitless injection mode, using a constant flow of helium gas (1 ml/min). The column oven temperature started at 50 °C and 1-min hold, increased to 300 °C at a rate of 20 °C/min from 1 to 13.5 min, followed by 8.5 min hold at 300 °C. The total run time was 22 min and electron ionization (70 eV) was used to generate the ions of metabolites for mass spectrometric detection.

## Results

### Microsomal metabolism of phenanthrene and its alkylated congeners

**Fig. 2:**
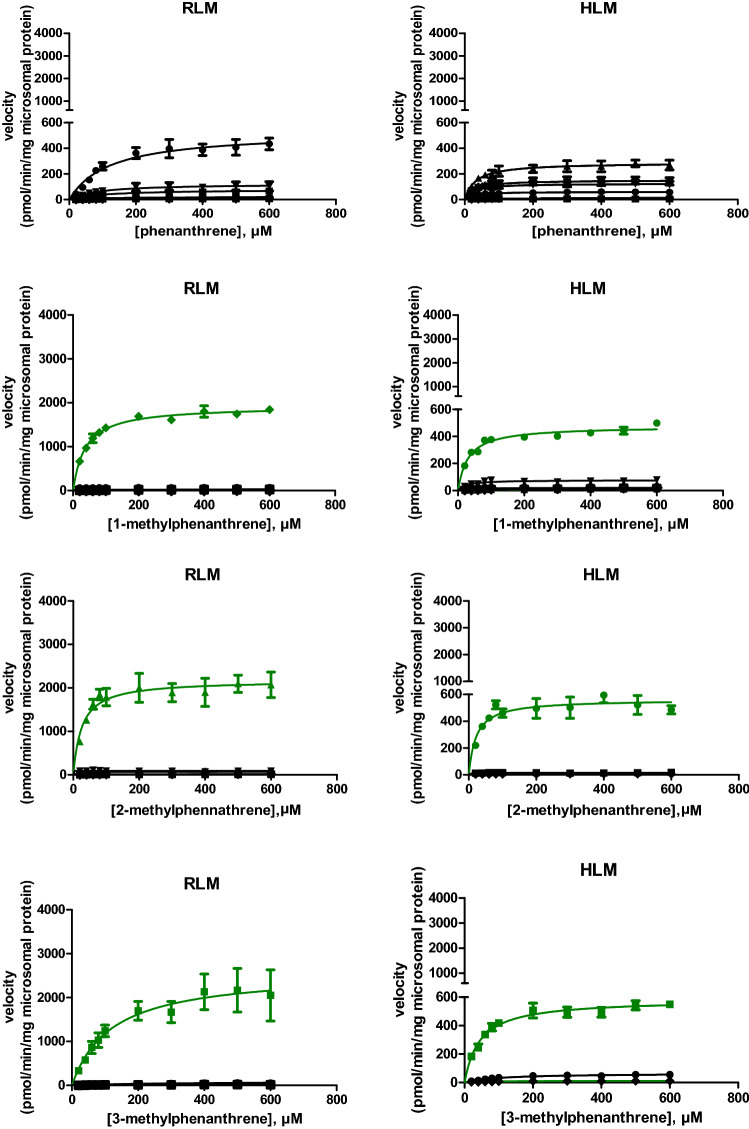

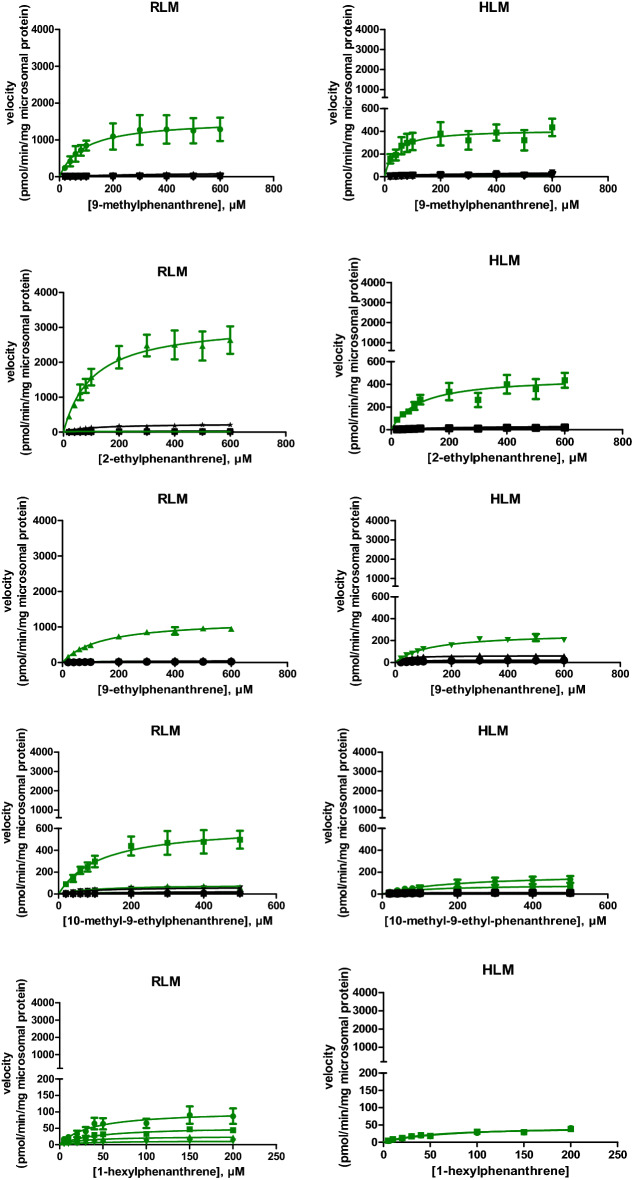
Substrate concentration-dependent metabolism of alkyl-substituted phenanthrenes and phenanthrene itself by RLM and HLM. Green lines represent metabolite formation via alkyl chain oxidation and black lines present metabolite formation by aromatic ring oxidation. Each symbol represents experimental means and vertical bars are standard errors of the mean (*n* = 3) (color figure online)

The concentration-dependent rate of metabolite formation and the corresponding fitted curves representing Michaelis–Menten kinetics of the metabolite formation mediated by RLM and HLM are shown in Fig. 2 for the model compounds. The tested compounds were phenanthrene, 1-methylphenanthrene, 2-methylphenanthrene, 3-methylphenanthrene, 9-methylphenanthrene, 2-ethylphenanthrene, 9-ethylphenanthrene, 10-methyl-9-ethyl-phenanthrene and 1-n-hexylphenanthrene. No metabolic conversion was observed for 1-n-dodecyl-phenanthrene under the experimental conditions used. The obtained *K*_*M*_ and *V*_max_ values and the calculated Cl_int_ for formation of each metabolite derived from the curves presented in Fig. 2 are summarized in Table 1.

Dihydro-phenanthrene-diols and phenanthrols were detected in both rat and human liver microsomal incubations of phenanthrene. Specifically, three major dihydro-phenanthrene-diols, 3,4-dihydro-phenanthrene-diol, 9,10-dihydro-phenanthrene-diol, and 1,2-dihydro-phenanthrene-diol, were characterized based on comparison with reference materials and the available literature (Bao and Yang 1991; Chaturapit and Holder 1978; Jacob et al. 1996; Schober et al. 2010; Shou et al. 1994; Sims 1970). For 3,4-dihydro-phenanthrene-diol and 9,10-dihydro-phenanthrene-diol, two partially overlapping peaks, representing their respective *cis-* and *trans-*isomers, were detected at 2.13 and 2.24 min and at 3.28 and 3.38 min, respectively. Three phenanthrols were identified as 3-phenanthrol, 1-phenanthrol, and 4-phenanthrol by co-elution and identical UV spectra with the reference standards.

The oxidative metabolism of alkyl-substituted phenanthrenes primarily occurred on the alkyl chain. The most abundant type of metabolites detected in both rat and human liver microsomal incubation mixtures with the alkyl-substituted phenanthrenes were alcohols. The primary metabolites of 3-methylphenanthrene and 9-methylphenanthrene were identified as 3-hydroxymethyl-phenanthrene and 9-hydroxymethyl-phenanthrene, respectively, which co-eluted with the reference standards in both UPLC and GC–MS/MS analyses. The molecular ion and base peak of both 3-hydroxymethyl-phenanthrene and 9-hydroxymethyl-phenanthrene were observed at m/z 208 and m/z 179, respectively. A comparable mass spectrum was obtained for the primary metabolite of 1-methylphenanthrene with molecular ion at m/z 208 and a base peak at m/z 179 supporting its identification as 1-hydroxymethyl-phenanthrene with an identical UV spectra as reported for this compound (Huang et al. 2017). With a similar mass spectral profile and elution pattern, 2-hydroxymethyl-phenanthrene was identified as a primary metabolite of 2-methylphenanthrene. 2-(1-Hydroxyethyl)-phenanthrene and 9-(1-hydroxyethyl)-phenanthrene were found to be the primary metabolites of 2-ethylphenanthrene and 9-ethylphenanthrene, respectively, with a molecular ion at *m*/*z* 222 and a base peak at *m*/*z* 179. In analogy with these results, the most abundant metabolite of 10-methyl-9-ethylphenanthrene was tentatively identified as 10-methyl-9-(1-hydroxyethyl)-phenanthrene with a base peak at *m*/z 203, a molecular ion peak at *m*/*z* 236 and the presence of other ion peaks at *m*/*z* 179 and *m*/*z* 218.

For alkylated phenanthrenes, dihydro-phenanthrene-diols and phenanthrols were minor metabolites formed in microsomal incubations with both RLM and HLM. On UPLC the most polar dihydrodiols eluted first, followed by alcohols, and subsequently by less polar phenanthrols before the parent compound eluted (Table 1), whereas on GC–MS/MS the parent compound with lowest boiling point eluted before the alcohols, dihydrodiols and phenanthrols. Identification of the minor metabolites was based on comparison with reference mass spectra in the NIST libraries or mass spectra of a reference compound that shared a similar structure. The detailed identification of the different microsomal metabolites formed from the alkyl-substituted phenanthrenes can be found in supplementary material 2.

### Intrinsic clearance by aromatic and alkyl side chain oxidation

**Fig. 3:**
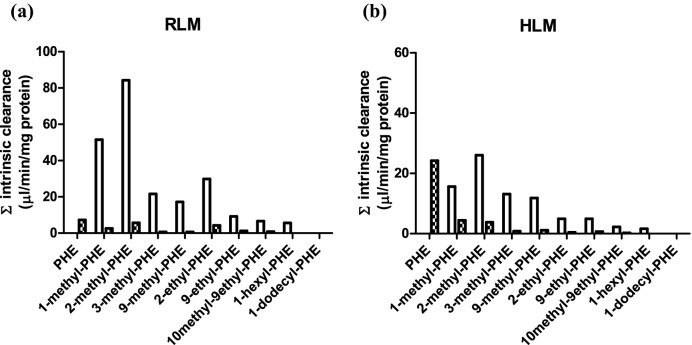
Intrinsic clearance via aromatic ring and alkyl chain oxidation by **a** RLM and **b** HLM for the different model compounds. Dashed bars represent aromatic ring oxidation; white bars represent alkyl chain oxidation. Abbreviation. PHE = phenanthrene

To compare the metabolic efficiency of alkyl side chain oxidation and aromatic ring oxidation of phenanthrene and its alkyl-substituted analogs, the intrinsic clearance of each parent test compound via side chain metabolites and aromatic ring metabolites was calculated (Fig. 3). The overall intrinsic clearance of phenanthrene was 7.3 and 24.2 µl/min/mg protein when metabolized by hepatic microsomes of rat and human, respectively. With increasing chain length the intrinsic clearance decreased substantially with for phenanthrenes with an alkyl side chain with more than three carbon atoms clearance being limited or even (1-n-dodecylphenanthrene) not observed at all (Fig. 3). The results presented in Fig. 3 also reveal that when phenanthrene was alkyl-substituted aromatic ring oxidation was reduced in favor of alkyl side chain oxidation. When there were more than 3 carbon atoms in the alkyl side chain of the tested substrates, aromatic ring oxidation was no longer detectable. In case of HLM, the intrinsic clearance of the tested alkylated phenanthrenes for which aromatic ring oxidation was observed was 5.5–121 times lower than that of phenanthrene. For metabolism by HLM of the tested substrates with up to three carbon atoms in the alkyl side chain, the intrinsic clearance via alkyl side chain oxidation was 3.5–16.4 times greater than that via aromatic ring oxidation. RLM metabolized the alkylated phenanthrenes via side chain oxidation 1.6–6.1 times more efficiently than HLM. For RLM the intrinsic clearance via aromatic ring oxidation of the tested alkylated phenanthrenes was 1.3- to 10.4-fold lower than that of phenanthrene itself. For the alkylated phenanthrenes for which aromatic oxidation was observed, the alkyl side chain oxidation was 7.0–30.8 times more efficient than the aromatic ring oxidation in microsomal incubations with RLM.

### Effect of alkyl chain length on total intrinsic clearance

**Fig. 4:**
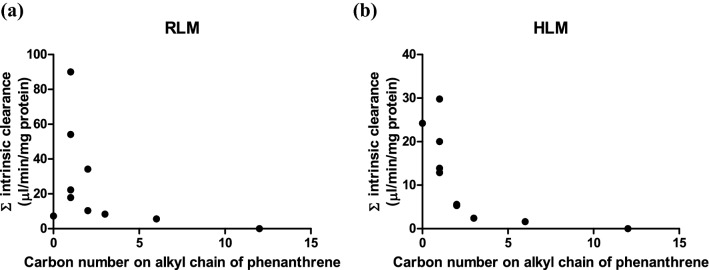
Relationship between total Cl_int_ of alkylated phenanthrenes and the number of carbon atoms in the alkyl side chain in metabolism with **a** RLM and **b** HLM. The intrinsic clearance was calculated by adding up the Cl_int_ values of all metabolites for the respective model compound (Table 1)

By adding up the intrinsic clearance of all metabolites for each substrate, the total intrinsic clearance of phenanthrene with and without alkyl substitution based on side chain carbon number was calculated. Figure 4 presents this overall intrinsic clearance via aromatic ring and side chain oxidation as a function of the number of carbon atoms in the alkyl side chain for the different model compounds. The results thus obtained show that the metabolism of alkylated phenanthrenes by both RLM and HLM becomes less efficient with elongation of the alkyl chain, especially when the side chain carbon number was more than 3. No metabolic conversion was detected when the side chain carbon number was 12.

### Mutagenicity of phenanthrene and its methylated substituents in the Ames test

**Fig. 5:**
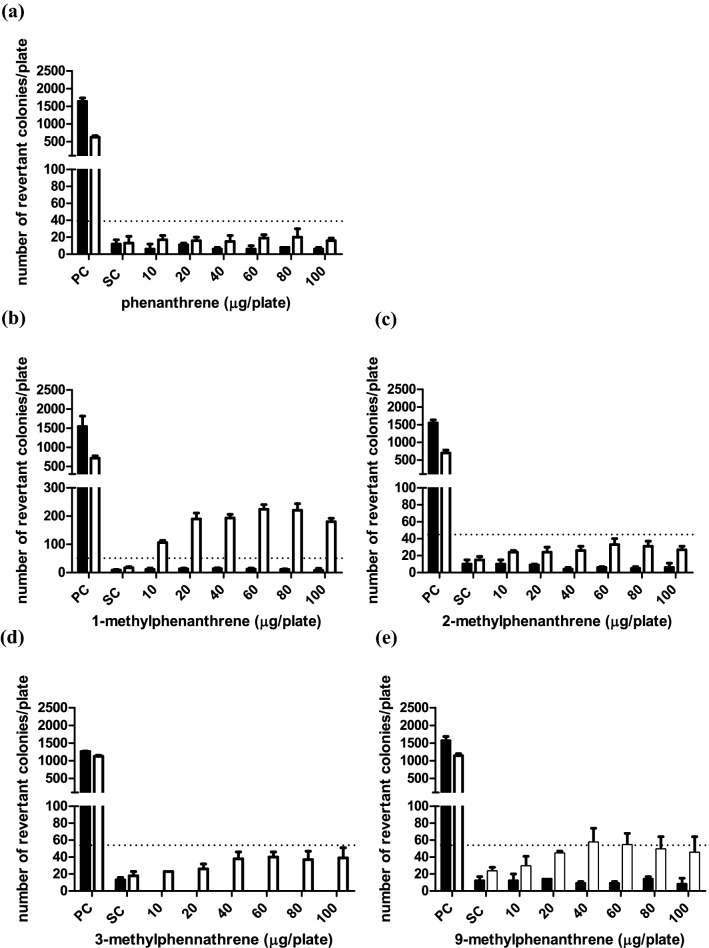
Number of revertants in *S. typhimurium* TA98 upon exposure to **a** phenanthrene, **b** 1-methylphenanthrene, **c** 2-methylphenanthrene, **d** 3-methylphenanthrene and **e** 9-methylphenanthrene in absence (black bar) and presence (white bar) of 5% S9-mix. Bars represent means and vertical bars indicate the standard deviation of the mean (*n* = 3). The dotted horizontal line indicates threefold increase that is considered the threshold for concluding on the positive outcome for mutagenicity. In the absence of S9 mix, the test doses of 3-methyl-phenanthrene showed cytotoxicity so no data are presented. The results of at least four analyzable doses that were non-cytotoxic are presented in Table S2 in the supplementary material 1 showing negative results. *PC* positive control, 1 µg/plate 2AA with S9-mix and 10 µg/plate NF without S9-mix. *SC* solvent control, DMSO with and without S9-mix

**Fig. 6:**
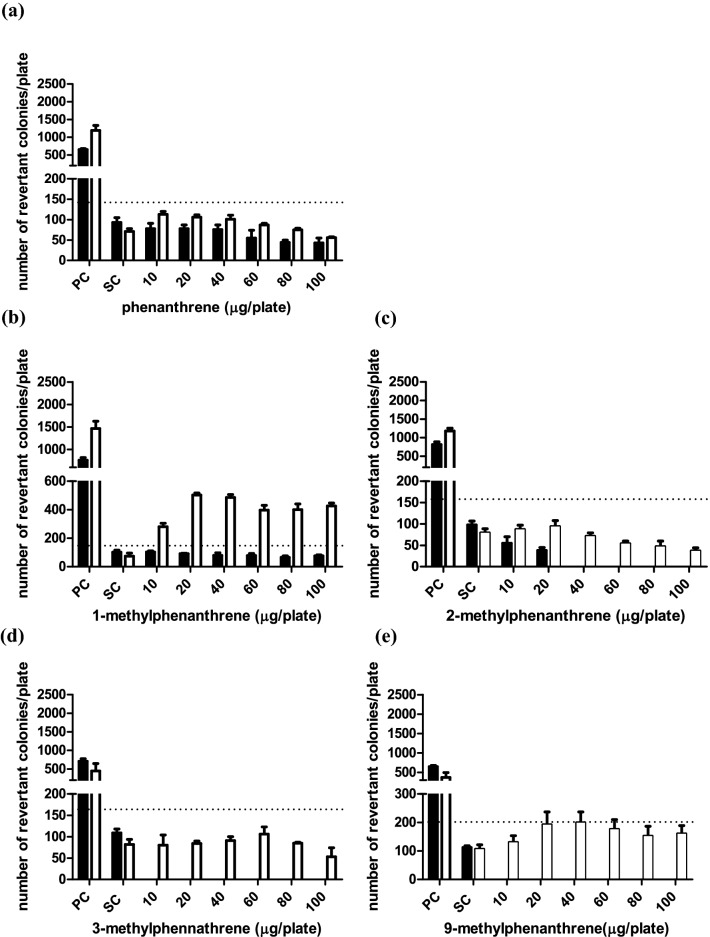
Number of revertants in *S. typhimurium* TA100 upon exposure to **a** phenanthrene **b** 1-methylphenanthrene, **c** 2-methylphenanthrene, **d** 3-methylphenanthrene and **e** 9-methylphenanthrene in the absence (black bar) and presence (white bar) of 5% S9-mix. The dotted horizontal line indicates twofold increase that is considered the threshold for concluding on the positive outcome for mutagenicity. Bar represents means and vertical bars are standard deviation of the mean (*n* = 3). In the absence of the S9 mix, the test doses of 2-methylphenanthrene, 3-methylphenanthrene and 9-methylphenanthrene showed cytotoxicity so no data are presented. The results of at least four analyzable doses that were non-cytotoxic are presented in Table S3 in the supplementary material 1 showing negative results. *PC*  positive control, 5 µg/plate 2AA with S9-mix and 650 µg/plate MMS without S9-mix. *SC*  solvent control, DMSO with and without S9-mix

The reverse mutation assay (Ames test) was used to assess the effect of methylation of phenanthrene on its mutagenicity toward *S. typhimurium* tester strains TA98 and TA100. The model compounds used for these studies were non-substituted phenanthrene, phenanthrene with the methyl substitution at the C1 or C9 position generating a “fake” bay region and at C2 and C3 for which this was not the case. No precipitation was observed on the plates at the tested concentrations under the microscope. None of the test compounds showed mutagenic potential in both tester strains in the absence of S9 metabolic system (Figs. 5 and 6). Figures 5 and 6 also present the number of (His ^+^) revertant colonies per plate induced in the presence of an S9 metabolic system by (a) phenanthrene, (b) 1-methylphenanthrene, (c) 2-methylphenanthrene, (d) 3-methylphenanthrene and (e) 9-methylphenanthrene for tester strain TA98 and TA100, respectively. No increases in the number of revertants were observed in TA 98 and TA100 upon exposure to phenanthrene in the presence of an S9 metabolic system. The observed increases in the number of revertants upon exposure to 1-methylphenanthrene in the presence of an S9 metabolic activation system was up to 13- and 6.8-fold compared to the concurrent solvent control for tester strain TA98 and TA100, respectively. In the presence of S9 metabolic activation, for both 2-methylphenanthrene and 3-methylphenanthrene, a slight dose response with 2.2-fold increase was observed in TA98; however, this was lower than the 3-fold increase required to conclude on a positive response that is biologically relevant (Levy et al. 2019) and no increased response was found in TA100 for either compound. Therefore, 2-methyl- and 3-methyl- phenanthrene were considered to be non-mutagenic in both TA98 and TA100. 2.5-Fold and 1.9-fold increases in the number of revertants compared to the concurrent solvent control were observed for 9-methyl-phenanthrene in TA98 and TA100, respectively, which were both lower than the 3-fold and 2-fold criteria. Close inspection of the corresponding solvent control data revealed that this was caused by the relatively high number of revertants in the concurrent solvent controls in the TA98 and TA100 assay of 9-methylphenanthrene, e.g., 23 ± 5 for the solvent control of 9-methylphenanthrene compared to the historical dataset with a value of 18 ± 6 for TA98 (Table S1). This implies that relative to the historical control data the increase in the number of revertants of 9-methyl-phenanthrene are 3.2-fold and 2.0-fold and are considered to be biologically relevant and indicative of mutagenicity.

### S9 mediated metabolism of phenanthrene and its methylated analogs

**Fig. 7:**
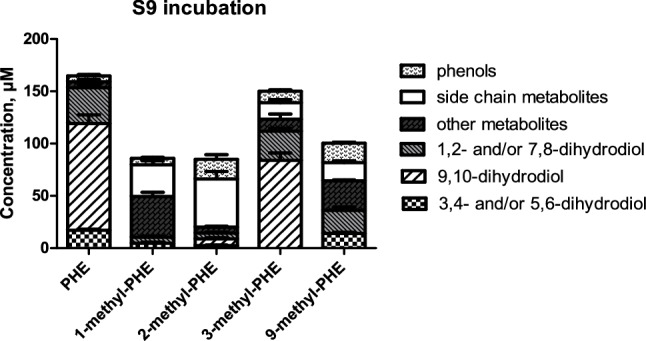
Concentration of the metabolites formed in S9 incubation with phenanthrene and four of its methyl-substituted analogs. Each bar represents experimental means and vertical bars are standard errors of the mean (*n* = 3)

To obtain insight into the metabolic activation of the four selected methyl-substituted phenanthrenes tested in the reverse mutation assay, exposure mixtures similar to those of the Ames test were analyzed by UPLC and further by GC–MS/MS for the unidentifiable metabolites. Figure 7 shows the metabolite patterns and quantification from the incubations with phenanthrene and its four methylated phenanthrenes (tested at 1000 µM a concentration being equivalent to the highest dose of 100 µg tested in the Ames test). Similarly to the results obtained with the microsomal incubations, metabolism of methylated phenanthrenes mediated by aroclor 1254 induced S9-mix generated dihydrodiols, alcohols and phenols (Figure S1 in supplementary material 1). This reveals that similar to what was already observed for the microsomal incubations, also for the S9 incubations the presence of the alkyl substituent shifts the metabolism of phenanthrene in favor of side chain oxidation at the cost of aromatic oxidation. Besides, an additional type of metabolite (reflected by a peak marked with an asterisk in Figure S1 supplementary material 1) was identified as hydroxymethyl-hydroxy-phenanthrene representing a further metabolite of hydroxymethyl-phenanthrene with a molecular ion at m/z 226 measured by GC–MS/MS.

Three major metabolites of phenanthrene observed in the chromatogram (Figure S1 in supplementary material 1) of the S9 incubation were identified as 3,4-dihydrodiol, 9,10-dihydrodiol and 1,2-dihydrodiol accounting for 10.5%, 62.1% and 20.7% of the total metabolite formation of phenanthrene, respectively. Formation of 1,2- and/or 7,8-dihydrodiols was found in incubations with all tested monomethylated phenanthrenes. Variation in the formation of 3,4- and/or 5,6-dihydrodiols and 9,10-dihydrodiol between mutagenic phenanthrenes (1-methyl- and 9 methyl-phenanthrene) and non-mutagenic phenanthrenes (2-methyl- and 3-methylphenanthrene) was noticed (Fig. 7). Formation of 9,10-dihydrodiol was only found in the incubations with 2-methyl and 3-methylphenanthrene at levels amounting to 7.7% and 57.7% of the metabolites formed, respectively. In the S9 incubations, 3,4- and/or 5,6-dihydrodiols were formed at levels amounting to 5.5% and 12.5% of the total metabolite formation for 1-methyl- and 9-methyl-phenanthrene, and at 2.2% and 0% of the total metabolite formation for 2-methyl and 3-methylphenanthrene, respectively.

## Discussion

The consumption of food contaminated with PPAHs from petroleum-derived products or environmental sources may be of concern to human health. The hazards of PPAHs are expected to be related to bioactivation. Since data on the metabolic fate of most PPAHs, in particular alkylated PAHs, are lacking, the microsomal oxidative metabolism, likely mediated by cytochrome P450 enzymes, of phenanthrenes with various types of alkylation was studied to investigate the effect of the alkyl substitution. It was hypothesized that alkylation of phenanthrenes would shift metabolism to alkyl side chain oxidation at the cost of aromatic ring oxidation when compared to non-alkylated phenanthrene. This metabolic shift would be expected to lower the chances of bioactivation to metabolites such as possibly mutagenic dihydrodiols and developmental toxicity related phenols. In line with the hypothesis, the side chain hydroxylated metabolites of alkylated phenanthrenes were 30.9- to 3.5-fold more efficiently formed than aromatic ring oxidation metabolites by both RLM and HLM. The overall metabolism of phenanthrene with an alkyl chain with more than six carbon atoms was strongly reduced and metabolism was even absent in the case of 1-n-dodecylphenanthrene (C12). This observation may be best ascribed to possible steric hindrance by the longer alkyl chains hampering binding to the active site of the cytochrome P450 enzymes. Furthermore, metabolic oxidation primarily happened on the carbon atom at the benzylic position in case of 2-ethyl-, 9-ethyl-, and 10-methyl-9-ethyl-phenanthrenes, similar to the results obtained by microsomal incubations of 1-ethyl- and 2-ethyl-naphthalene (Wang et al. 2020). Side chain oxidation of alkylated phenanthrenes was also observed in other metabolic studies. Hydroxymethyl-phenanthrenes were detected as primary metabolites in a metabolism study with rat S9 incubations of 1-methyl-, 2-methyl, 3-methyl-, 4-methyl- and 9-methyl-phenanthrene (LaVoie et al. 1981). Side chain hydroxylation metabolites were also detected in incubations of 1-methylphenanthrene and 9-ethylphenanthrene with human hepatoma (HepG2) cells (Huang et al. 2017).

Considering that the metabolic shift from aromatic ring to side chain oxidation may reflect a shift from bioactivation to detoxification, data on the mutagenicity and tumorigenicity of these alkylated PAHs are of interest. However, data on the mutagenicity and the tumorigenicity of phenanthrene and alkyl-substituted phenanthrenes are scarce. Neither phenanthrene nor its dihydrodiol metabolites were found to be mutagenic toward TA98 and TA100 tester strain of *S. typhimurium* (Bucker et al. 1979; Wood et al. 1979). This is in line with the observation in the present study that phenanthrene tested negative for mutagenicity in both tester strains TA98 and TA100. In the present study, 1-methylphenanthrene showed 13-fold and 6.8-fold increase in the number of revertants compared to the concurrent solvent control when tested in the presence of metabolic activation in TA98 and TA100, respectively. Comparable studies showed a 3.2-fold and 6.0-fold increase in TA98 and TA100, respectively (LaVoie et al. 1983), and an 8.4-fold and 4.1-fold increase in the same strains (Katarzyna Rudnicka et al. 2013). In addition, 9-methylphenanthrene, the other methyl-substituted phenanthrene that tested positive with 3.2-fold (TA98) and 2.0-fold (TA100) increase relative to historical controls in the present study, was previously reported to be mutagenic as reflected by a 3.0-fold increase in revertants in TA100 while testing negative (only 1.3-fold increase) in TA98 (LaVoie et al. 1983).

**Fig. 8:**
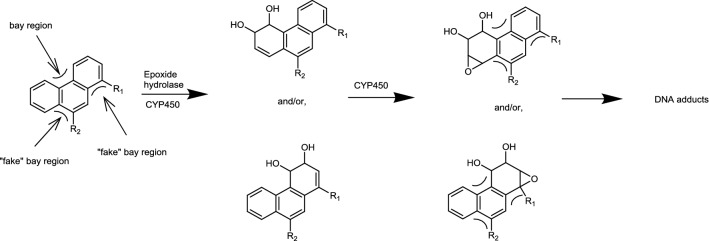
Possible metabolic pathway toward bay region dihydrodiol-epoxide formation for 1-methyl- (R_1_=CH_3_) and 9-methyl- (R_2_=CH_3_) phenanthrene

Considering that the possible metabolic pathway underlying mutagenicity of 1-methyl- and 9-methyl-phenanthrene could be a dihydrodiol-epoxide pathway (Fig. 8), it can be suggested that the observed mutagenicity of 1-methyl- and 9-methyl-phenanthrene may be associated with regio- and stereo- selectivity for the possible dihydrodiol-epoxide bioactivation following the introduction of the methyl substitution. The formation of an additional bay region-like structural motif, described as “fake” bay region (Fig. 8) upon introduction of a methyl substituent at the C1 or C9 position of phenanthrene, may play a role in the observed mutagenicity. It is also of interest to note that it has been suggested before that the formation of 3,4- and/or 5,6- dihydrodiol metabolites likely reflects the bioactivation to mutagenic metabolites (LaVoie et al. 1981). This would be in line with the suggestion that methyl substitution at or near the K-region (9,10-position) of phenanthrene at its peri positions (Fig. 1) would favor 3,4- and/or 5,6-dihydrodiol formation and mutagenicity (Fig. 5 and 6) (LaVoie et al. 1983). Indeed for 9-methyl- and 1-methyl-phenanthrene, formation of 3,4- and/or 5,6-dihydrodiol was observed, albeit to a level somewhat lower than that observed for phenanthrene itself which was tested negative for mutagenicity. Nevertheless, given the fact that methyl substitution adjacent to the K-region in 1-methyl- and 9-methyl phenanthrene forms a “fake” bay region, it implies that 3,4- and/or 5,6-dihydrodiol formation provides increased chances of formation of a bay-region dihydrodiol-epoxide (Fig. 8). However, in phenanthrene this 3,4- and/or 5,6-dihydrodiol formation would not result in a bay region dihydrodiol-epoxide.

Given these mutagenicity results, it is also important to consider that neither phenanthrene nor its monomethylated analogs, including 2-methyl- and 3-methyl-phenanthrene but also 1-methyl- and 9-methyl-phenanthrene, were found to be active tumor initiators in mouse skin painting studies (Buening et al. 1979; LaVoie et al. 1981). This lack of tumor-initiating activity of methyl-substituted phenanthrenes might be due to a relatively lower tumor inducing potency of the formed DNA adducts compared to for example the dihydrodiol-epoxide metabolites of benzo[a]pyrene.

Finally, it is also of interest that some authors have proposed a role for side chain hydroxylated metabolites in alkyl-substituted PAHs to play a role in their mutagenicity upon their further bioactivation by sulfotransferases to unstable DNA reactive sulfate metabolites (Huang et al. 2017). Whether for 1-methyl- and 9-methyl-phenanthrene also their side chain hydroxy metabolites play such a role remains to be investigated.

The current risk assessment of PPAHs that may be present in consumer products is based on read across to metabolic activation and formation of DNA reactive metabolites of naked PAHs due to the lack of data on PPAH themselves (Baird et al. 2007; EFSA 2012; Wickliffe et al. 2014). The results of the present study provide insight in the effect of alkylation on the oxidative metabolism of phenanthrene, and also provide kinetic parameters that may turn out to be of use for future physiologically based kinetic (PBK) models to extrapolate toxicity data obtained in vitro to in vivo taking kinetics into account. The PBK models together with in vitro concentration–response data could provide the basis for a new approach methodology (NAM) for predicting the in vivo toxicity of alkyl-substituted aromatics that may be present in petroleum-derived products, in line with the proof of principle predicting the developmental toxicity of benzo[a]pyrene by this in vitro–in silico approach (Wang et al. 2021).

Phenanthrene is the smallest PAH with a bay region, but lacks genotoxic and carcinogenic properties observed for some bay-region PAHs with a higher number of aromatic rings, such as benzo[a]pyrene. It would be of interest for future studies to investigate whether alkylation causes similar shifts in metabolic oxidation for PAHs and PPAHs with more fused aromatic rings and in what way this influences their toxicity, as observed with naphthalene (Wang et al. 2020) and with phenanthrene in the present study. The results of the present study clearly show that the mutagenic effect depends on the site of alkylation on PAHs.

Taking all together, it is concluded that alkylation of PAHs favors alkyl chain oxidation at the cost of aromatic oxidation. Especially methyl substitution of phenanthrene adjacent to its K-region such as in 1-methyl- and 9-methyl-phenanthrene converted phenanthrene into mutagens toward *S. typhimurium* TA98 and TA100. The position of the alkylation affects the metabolism and resulting mutagenicity of phenanthrene with the mutagenicity increasing in cases where the alkyl substituent creates an additional bay region-like structural motif, in spite of the extra possibilities for side chain oxidation.

## Supplementary Information

Below is the link to the electronic supplementary material.

Supplementary file1 (DOCX 119 KB)

Supplementary file2 (DOCX 14 KB)
