# Supplementary material for: The effect of alkyl substitution on the oxidative metabolism and mutagenicity of phenanthrene
Source: Arch Toxicol. 2022 Feb 19;96(4):1109–31. doi: 10.1007/s00204-022-03239-9 (PMC8921064; doi:10.1007/s00204-022-03239-9)
Supplement: Supplementary file 1 — Supplementary file1 (DOCX 119 KB) [file 204_2022_3239_MOESM1_ESM.docx]

Table S1 Historical control data of the solvent control of reverse mutation assay

|  | TA98 | | TA100 | |
| --- | --- | --- | --- | --- |
| S9-mix | - | + | - | + |
| Range | 4 – 61 | 6 – 60 | 58 – 188 | 50 – 176 |
| Mean | 14 | 18 | 109 | 101 |
| SD | 5 | 6 | 19 | 21 |
| n | 2523 | 2528 | 2566 | 2508 |

SD = Standard deviation

n = Number of observations

Historical control data from experiments performed between Nov 2017 and Nov 2020.

Table S2 Mutagenic response of 3-methyl-PHE in the Salmonella typhimurium TA98 without S9-mix at lower doses that were non-cytotoxic. The results are presented as mean number of revertant colonies of 3 replicate plates and standard deviation.

| Test compound | Dose (µg/plate) | His + revertant colonies TA98  without S9-mix |
| --- | --- | --- |
| 3-methyl-PHE | PC | 1590±295 |
|  | SC | 15±4 |
|  | 2 | 16±3 |
|  | 4 | 17±4 |
|  | 10 | 14±1 |
|  | 20 | 13±2 |
|  | 40 | 9±4^S^ |

^S^ indicates bacterial background lawn slightly reduced

Table S3 Mutagenic response of 2-methyl-PHE, 3-methyl-PHE and 9-methyl-PHE in the Salmonella typhimurium TA100 without S9-mix at lower doses that were non-cytotoxic. The results are presented as mean number of revertant colonies of 3 replicate plates and standard deviation.

| Test compound | Dose (µg/plate) | His + revertant colonies TA100  without S9-mix |
| --- | --- | --- |
| 2-methyl-PHE | PC | 823±58 |
|  | SC | 97±4 |
|  | 10 | 106±20 |
|  | 20 | 98±13 |
|  | 40 | 70±8^m^ |
| 3-methyl-PHE | PC | 783±46 |
|  | SC | 116±9 |
|  | 2 | 112±14 |
|  | 4 | 112±9 |
|  | 10 | 119±11 |
|  | 20 | 83±34 |
|  | 40 | 75±4^m^ |
| 9-methyl-PHE | PC | 868±70 |
|  | SC | 115±17 |
|  | 2 | 121±7 |
|  | 4 | 109±3 |
|  | 10 | 105±11 |
|  | 20 | 97±20 |
|  | 40 | 59±40^m^ |

^m^ inducates bacterial background lawn moderately reduced


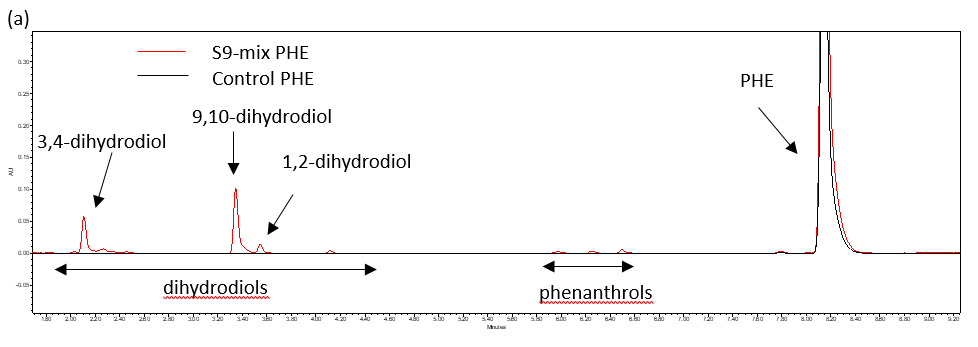


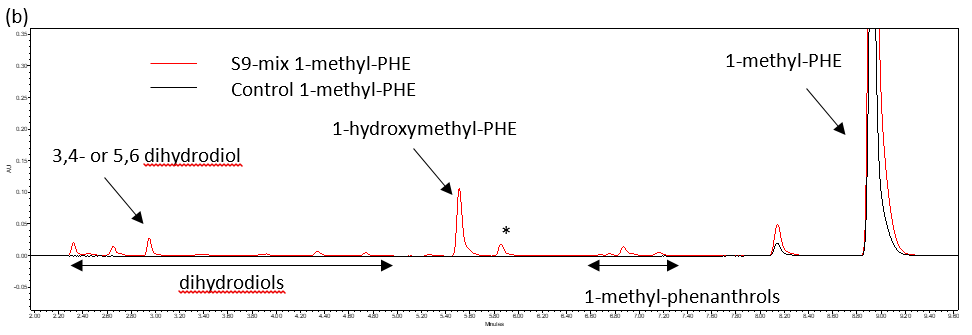


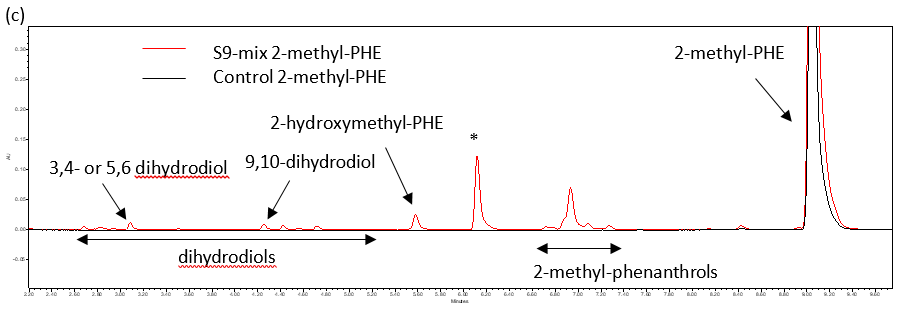


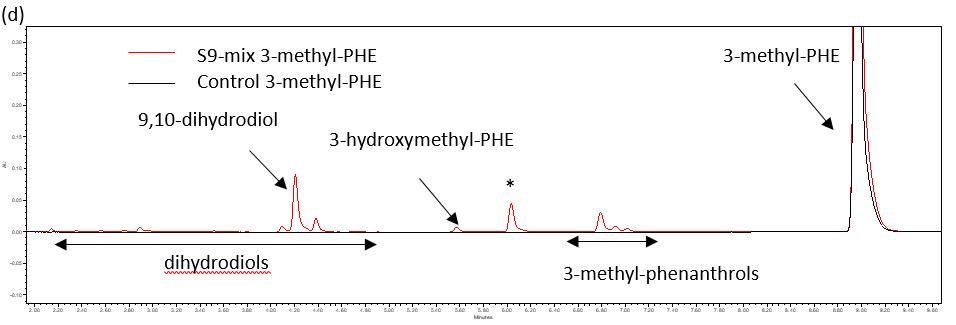


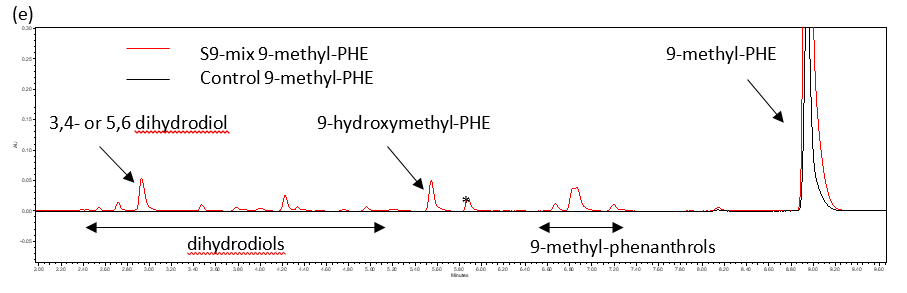


Figure S1. Relevant parts of UPLC chromatograms of incubation mixtures without and with S9-mix for (a) phenanthrene (b) 1-methylphenanthrene (c) 2-methylphenanthrene (d) 3-methylphenanthrene and (e) 9-methylphenanthrene at 254 nm wavelength. PHE = phenanthrene.
