# Supplementary material for: The effect of alkyl substitution on the oxidative metabolism and mutagenicity of phenanthrene
Source: Arch Toxicol. 2022 Feb 19;96(4):1109–31. doi: 10.1007/s00204-022-03239-9 (PMC8921064; doi:10.1007/s00204-022-03239-9)
Supplement: Supplementary file 2 — Supplementary file2 (DOCX 14 KB) [file 204_2022_3239_MOESM2_ESM.docx]

Four dihydrodiols of 2-methylphenanthrene were detected at retention time 3.09 min, 4.26 min, 4.42 min and 4.99 min ionized to a molecular ion at m/z 226 and a base peak at m/z 165. The dihydrodiols eluting at 3.09 min and 4.26 min were identified as 3,4- and 9,10- dihydro-2-methylphennathrene-diol based on both the retention time and comparison to the UV spectra of 3,4-dihydro-phenanthrene-diol and 9,10-dihydro-phennathrene-diol. Three phenolic metabolites eluting at 6.12 min, 6.98 min and 7.08 min were identified as 2-methylphenanthrols.

The metabolites of 3-methylphenanthrene at retention time 4.25 min and 4.42 min were identified as dihydro-3-methylphenanthrene-diols with a molecular ion at m/z 226 and a base peak at m/z 165. The dihydrodiol eluting at 4.25 min was identified as 9,10-dihydro-3-methylphennathrene-diol based on both the retention time and comparison to the UV spectrum of 9,10-dihydro-phenanthrene-diol. The metabolite of 3-methylphenanthrene eluting at 6.98 min was identified as 3-methyl-phenanthrol with a base peak at m/z 208.

The metabolite of 9-methylphenanthrene eluting at 2.62 min was identified as a dihydro-9-methylphenanthrene-diol with a molecular ion at m/z 226, most probably being the 3,4-dihydro-9-methylphenanthrene-diol based on both the retention time and comparison to the UV spectrum of 3,4-dihydro-phenanthrene-diol. An additional dihydrodiol was detected at retention time 4.27 min that was possibly 1,2-dihydrodio-9-methylphenanthrene-diol based on both the retention time and comparison of its UV spectrum to the UV spectrum of 1,2-dihydro-phenanthrene-diol. Five metabolites with retention times ranging from 6.28 to 7.25 min were identified as 9-methylphenanthrols with a base peak at m/z 208 and an ion peak at m/z 165.

The concentrations of the minor metabolites that were formed from 1-methylphenanthrene were too low to be quantified within the background noise of the GC-MS/MS measurements. However, based on the retention time of the metabolites formed from 3- and 9-methylphenanthrene, these minor metabolites could be tentatively identified as dihydro-1-methylphenanthrene-diols based on their retention times of 2.32 min, 3.43 min, 3.96 min, 4.35 min and 4.76 min. The metabolites formed from 1-methylphenanthrene eluting at 6.3 min to 7.29 min were tentatively assigned as 1-methylphenanthrols.

The mass spectra of the 2-ethylphenanthrene metabolites at retention times of 4.6 min, 5.16 min and 5.29 min all showed a molecular ion at m/z 238 and a base peak at m/z 165, and could therefore be identified as dihydro-2-ethylphenanthrene-diols. The metabolites at 7.57 min, 7.74 min and 7.89 min were identified as 2-ethylphenanthrols based on their molecular ion at m/z 222 in combination with a base peak at m/z 207. An additional metabolite that was tentatively identified as methyl-2-phenanthryl-ketone could not be found on UPLC but was detected by GC-MS/MS at a retention time of 12.03 min with a molecular ion at m/z 220 and a base peak at m/z 205, partly overlapping with the peak of 2-(1-hydroxyethyl)-phenanthrene at 12.00 min.

Minor metabolites of 9-ethylphenanthrene eluting at 3.81 min and 3.88 min were identified as dihydro-9-ethylphenanthrene-diols based on their molecular ions at m/z 238 and base peaks at m/z 165. The metabolite eluting at 6.00 min, shortly before 9-(1-hydroxyethyl)-phenanthrene was tentatively identified as 9-(2-hydroxyethyl)-phenanthrene based on a molecular ion at m/z 222 and a base peak at m/z 191. The metabolites at retention times of 7.42 min, 7.58 min and 7.93 min all had a molecular ion at m/z 222 and a base peak at m/z 207, and were identified as 9-ethylphenanthrols.

Two metabolites of 10-methyl-9-ethylphenanthrene, with retention times of 4.29 min and 4.34 min, were identified as dihydro-10-methyl-9-ethyl-phenanthrenediol with a molecular ion at m/z 254 and a base peak at m/z 167. The metabolite eluting at 6.5 min was identified as 10-methyl-9-(2-hydroxyethyl)-phenanthrene, an ethanol with a molecular ion at m/z 236 and a base peak at m/z 205. The other alcohol metabolite eluting at 6.90 min, was identified as 10-(1-hydroxymethyl)-9-ethylphenanthrene with a molecular ion at m/z 236 and a base peak at m/z 221. The metabolites at retention times 7.82 min and 8.30 min were identified as 10-methyl-9-ethyl-phenanthrols with a base peak at m/z 236.

Four metabolites of 1-n-hexylphenanthrene eluting from 8.45 min to 9.13 min were identified as 1-n-hydroxyhexyl-phenanthrenes based on the similarity of their UV spectra to the UV spectrum of 1-n-hexyl-phenanthrene, and the fact that they were ionized to a base peak at m/z 207 and showed a molecular ion at m/z 278.
